# *Clostridium perfringens* epsilon toxin binds to erythrocyte MAL receptors and triggers phosphatidylserine exposure

**DOI:** 10.1111/jcmm.15315

**Published:** 2020-05-28

**Authors:** Zhijun Geng, Jing Huang, Lin Kang, Shan Gao, Yuan Yuan, Yanwei Li, Jing Wang, Wenwen Xin, Jinglin Wang

**Affiliations:** ^1^ Graduate College Anhui Medical University Anhui China; ^2^ State Key Laboratory of Pathogen and Biosecurity Institute of Microbiology and Epidemiology AMMS Beijing China; ^3^ Life Science Institute of Hebei Normal University Shijiazhuang China

**Keywords:** ceramide, epsilon toxin, haemolysis, human erythroleukaemia cell line, myelin and lymphocyte receptor, phosphatidylserine

## Abstract

Epsilon toxin (ETX) is a 33‐kDa pore‐forming toxin produced by type B and D strains of *Clostridium perfringens*. We previously found that ETX caused haemolysis of human red blood cells, but not of erythrocytes from other species. The cellular and molecular mechanisms of ETX‐mediated haemolysis are not well understood. Here, we investigated the effects of ETX on erythrocyte volume and the role of the putative myelin and lymphocyte (MAL) receptors in ETX‐mediated haemolysis. We observed that ETX initially decreased erythrocyte size, followed by a gradual increase in volume until lysis. Moreover, ETX triggered phosphatidylserine (PS) exposure and enhanced ceramide abundance in erythrocytes. Cell shrinkage, PS exposure and enhanced ceramide abundance were preceded by increases in intracellular Ca^2+^ concentration. Interestingly, lentivirus‐mediated RNA interference studies in the human erythroleukaemia cell line (HEL) cells confirmed that MAL contributes to ETX‐induced cytotoxicity. Additionally, ETX was shown to bind to MAL in vitro. The results of this study recommend that ETX‐mediated haemolysis is associated with MAL receptor activation in human erythrocytes. These data imply that interventions affecting local MAL‐mediated autocrine and paracrine signalling may prevent ETX‐mediated erythrocyte damage.

## 1 INTRODUCTION

Epsilon toxin (ETX) synthesized by *C perfringens* types B and D is a key virulence factor.^1^ ETX causes a highly lethal enterotoxaemia that has a major impact on the rearing of domesticated ruminants, especially sheep.^2^ The 50% lethal dose of ETX in mouse is 100 ng/kg, which is the reported bacterial toxin that is second only to botulinum and tetanus toxins.^3^ ETX is classified as a potential category B biological weapon.^4^, ^5^

There are three domains in ETX: domain I is responsible for recognition and binding of receptors on host cells, domain II stabilizes binding of ETX to its receptor and triggers the formation of heptamers, and domain III is responsible for aggregation between ETX monomers to form pores in the cell membrane.^2^, ^6^, ^7^ The mechanisms and intracellular metabolic pathways associated with ETX‐induced cell death have not been well elucidated. The toxin induces cell changes associated with death, including the earliest changes in cell volume, followed by mitochondrial disappearance, cell membrane blistering and rupture, ATP release, nuclear size reduction, and increased propidium iodide (PI) uptake.^4^, ^8^, ^9^ The formation of pores in the affected cells leads to a rapid outflow of K^+^ in the cells, the inflow of Cl^‐^ and Na^+^, followed by an increase in intracellular ([Ca^2+^]_i_).^10^ Previously, we found that ETX is highly specific to human red blood cells, but does not cause haemolysis of erythrocytes in other species (murine, rabbit, sheep, goat, cattle, equine, dog, monkey).^11^ This finding prompted us to further study the mechanisms of ETX‐induced haemolysis.

Some bacterial toxins cause erythrocyte haemolysis through cell shrinkage, membrane blebbing and exposure of phosphatidylserine (PS) at the cell surface.^12^ These include *Escherichia coli* α‐haemolysin (HlyA),^13^ *Pseudomonas aeruginosa* pyocyanin^14^ and listeriolysin.^12^ The MAL receptor was found to be required for ETX cytotoxicity in oligodendrocytes,^15^ human T lymphocytes^16^ and polarized epithelial cells.^17^, ^18^ The relative simplicity of erythrocytes makes these cells a suitable model for addressing the basic mechanisms of ETX‐induced cell damage. Here, we investigated the role of MAL receptors in ETX‐mediated toxicity and lysis of human erythrocytes. Our results showed that ETX initially causes a significant decrease in erythrocyte size, followed by an increase in cell volume leading to lysis. Moreover, ETX insertion caused an increase in [Ca^2+^]_i_, enhanced ceramide abundance and promoted PS exposure in the outer leaflets of erythrocyte membranes. We also found that ETX‐mediated death of HEL cells requires MAL and that ETX was shown to bind to MAL in vitro. Together, these data suggest that MAL receptors play an important role in ETX‐mediated haemolysis.

## 2 MATERIALS AND METHODS

### 2.1 Materials

Anti‐MAL polyclonal antibody (reactivity: mouse, rat, dog, human, frog), anti‐ceramide polyclonal antibody, horseradish peroxidase (HRP)‐coupled goat antimouse IgG (H + L) antibody, anti‐His monoclonal antibody and fluorescein isothiocyanate (FITC)‐conjugated goat anti‐rabbit IgG (H + L) were purchased from Abcam (Cambridge, MA, USA). 3‐(4, 5‐dimethylthiazol‐2‐yl)‐5(3‐carboxymethoxyphenyl)‐2‐(4‐sulfopheny)‐2H‐tetrazolium inner salt (MTS) was purchased from Promega Corporation (Madison, WI, USA). Anti‐glutathione S‐transferase (GST) monoclonal antibody was purchased from EARTHOX Life Sciences (Millbrae, CA, USA). Annexin V, annexin V binding buffer and PE anti‐human CD235a (Glycophorin A) antibody were purchased from BioLegend (San Diego, CA, USA). Fluo‐4 and PKH26 Red Fluorescent Cell Linker Kit were purchased from Sigma (St. Louis, MO, USA). BAPTA‐AM, Protease inhibitor and 2ʹ,7ʹ‐Dichlorofluorescin Diacetate were purchased from Sigma (St. Louis, MO, USA).

### 2.2 Preparation of erythrocytes

Human blood was collected from healthy volunteers by venipuncture into evacuated blood collection tubes containing ethylenediaminetetraacetic acid‐2K. Erythrocytes were washed three times with 0.01 M phosphate‐buffered saline (PBS) (1000 × g, 4°C, 5 min). The serum layer was removed, and the pellet was the red blood cells.

### 2.3 Preparation of recombinant toxins

We constructed the recombinant plasmid vectors pTIG‐His‐ETX/pGEX‐GST‐ETX and pTIG‐mScarlet‐ETX‐His, encoding 6 × His/GST‐tagged ETX (without 22‐residue C‐terminal and 13‐residue N‐terminal sequences) and mScarlet‐ETX proteins, respectively. The both plasmids were transformed into *E coli* BL21 (DE3) cells. The transformed bacteria were grown in 5 mL of sterile lysogenic broth (LB) at 37°C for 6 hours with constant shaking (180 rpm). The cultures were transferred to 500 mL of sterile LB containing ampicillin (100 μg/mL) and grown for 4.5 hours at 37°C with constant shaking (180 rpm) until the exponential growth phase was reached (as assessed via OD_600_). Isopropyl β‐D‐1‐thiogalactopyranoside (0.5 mmol/L) was used to induce the expression of recombinant proteins overnight (16°C, 180 rpm). The following morning, the culture was centrifuged (3000 *g*) at 4°C for 5 minutes to precipitate bacteria.

To purify the His/GST‐tagged ETX proteins, the bacterial pellets were resuspended in lysis buffer and cells were lysed by sonication on ice. The lysates were centrifuged at 3300 *g* for 15 minutes at 4°C. The clarified supernatants were purified using a Ni^2+^/GST affinity chromatography column (GE Healthcare, Pittsburgh, PA, USA) as previously described. The purified proteins were analysed by 15% SDS‐PAGE. We selected purified toxins with a purity greater than 98% for subsequent experiments.

### 2.4 Measurements of haemolytic activity

The separated erythrocytes were diluted to a 5% solution with 0.01 M PBS. In the haemolysis test, purified ETX (different concentrations) was added to a 5% erythrocytes solution (final concentration of erythrocytes: 3.3%) and incubated at 37°C for 1 hour with continuous shaking (300 rpm). In other haemolysis experiments, different concentrations of inhibitor (BAPTA‐AM, GW4869, N‐oleoylethanolamine) were incubated at 37°C for 30 minutes, and purified ETX (0.2 μM) caused 50% haemolysis, incubate for 60 minutes at 37°C. The incubated erythrocytes were centrifuged at 1000 *g* for 10 minutes at 4°C, and the optical density at 540 nm of the supernatants was determined as a measure of the released haemoglobin. Relative haemolysis (values compared with a control, defined as 1) is shown in each figure; different controls are used in each figure. In general, maximal haemolysis in each figure was defined as 1(complete haemolysis caused by 10% Triton‐100).

### 2.5 Cell culture and treatment

The HEL cells were purchased from the China Infrastructure of Cell Line Resources. The cells were incubated in RPMI‐1640 medium contained with 10% FBS, which were cultured in a humidified chamber with 5% CO_2_. In order to interfere with the expression of MAL protein in HEL cells, lentiviral‐mediated RNA interference (control siRNA: TTCTCCGAACGTGTCACGTAA; mal siRNA: GACTTGCTCTTCATCTTTGAGTTTA) was packaged and synthesized by Hanbio Biotechnology (shanghai, china). HEL cells were seeded in 6‐well plates. When the cells grew to about 50%‐60%, lentiviral‐mediated RNA interference was added to the cells after replacing the new medium for 1 hours. After 3 days, 5 µg/ml of puromycin was added to screen for cells expressing lentiviral‐mediated RNA interference. Verification of MAL protein expression was performed after cell lines stably expressing lentiviral‐mediated RNA interference.

### 2.6 MTS assay

HEL cells (3‐4 × 10^4^ cells/well) were seeded in 96‐well plates and incubated at 37°C for 24 hours in a 5% CO_2_. Freshly purified toxin proteins were used in this assay. MTS assays were performed as described by the manufacturer.

### 2.7 Annexin V binding

For the study of PS exposure experiments, 10^5^ erythrocytes were analysed for each experiment. ETX (0.2 μM) was added to the cells and incubated for 1 hour at 37°C. The cells were harvested by centrifugation at 1000 *g* for 10 minutes, then resuspended in annexin V binding buffer containing annexin V and incubated for 10 minutes in the dark. The suspension was diluted fivefold in Ca^2+^‐containing saline and then analysed on a flow cytometer via 488‐nm excitation and 520‐nm emission.

### 2.8 [Ca^2+^]i imaging of human erythrocytes

Erythrocytes (~10^6^ cells/mL) were first incubated with Fluo‐4 AM (5 μM) for 25 minutes at 37°C in the dark, washed once with Ca^2+^‐containing buffer and then centrifuged at 1000 *g* for 10 minutes at room temperature. After addition of ETX (0.2 μM), the increase in fluorescence at 488 nm over time was measured.

### 2.9 Determine ceramide abundance

Ceramide concentrations in human erythrocytes were also assessed using the FACSaria. Erythrocytes (~10^6^ cells/mL) were preincubated for 1 hours with ETX (0.2 μM) at 37°C, washed once in Ca^2+^‐containing saline and centrifuged at 1000 × g for 10 minutes at room temperature. The cells were incubated for 2 hours with anti‐ceramide polyclonal antibody at 37°C, then washed once with Ca^2+^‐containing saline. After incubation with FITC‐conjugated secondary antibodies and washing once in Ca^2+^‐containing saline, fluorescence at 488 nm was measured.

### 2.10 Estimate reactive oxygen species (ROS)

To estimate the production of reactive oxygen species (ROS), erythrocytes were resuspended in Ringer's solution (125 mmol/L NaCl, 5 mmol/L KCl, 5 mmol/L glucose, 32 mmol/L HEPES, 1 mmol/L Mg_2_SO_4_, 1 mmol/L CaCl_2_, pH 7.4), and with non‐polar and non‐fluorescent probe 2ʹ,7ʹ‐dichlorofluorescin diacetate (10 μmol/L) was incubated for 30 minutes at 37°C. The cells were then washed and resuspended with Ringer's solution. The geometric mean of DCF‐dependent fluorescence was quantified using FACS analysis.

### 2.11 Determine MAL receptor and anti‐CD235a antibody expression

Erythrocytes (~106 cells/mL) were first incubated with anti‐MAL antibodies (1:100) for 1 hours at 37°C, washed once with PBS buffer and then centrifuged at 1000 *g* for 10 minutes at room temperature. The erythrocytes were incubated with PE‐conjugated anti‐human CD235a antibody and FITC‐conjugated secondary antibody at 37°C for 30 minutes, washed once with PBS buffer and then detected by flow cytometry. After flow cytometry, human erythrocytes were attached to glass slides and covered with cover slips. The slides were imaged under a laser confocal scanning microscope (SP8, LEICA).

### 2.12 Volume changes of human erythrocytes

For erythrocyte volume studies, 10^5^ cells were incubated with PKH26 Red Fluorescent Cell Linker. Human erythrocytes were stained according to the PKH26 Red Fluorescent Cell Linker Kit instructions. The cells were incubated with ETX (0.2 μmol/L) and quickly placed on glass slides. The slides were placed in a laser confocal scanning microscope for continuous imaging at 594 nm.

### 2.13 MAL protein detection by Western blotting

**MAL protein expression in human erythrocytes, rat erythrocytes, mouse erythrocytes, HEL cells and HEL‐∆MAL cells was assessed by Western blotting. The HEL and HEL‐∆MAL cells were seeded in a 10 cm diameter culture plate overnight; then, 1 × 10^6^ human, rat and mouse erythrocytes were taken and washed twice with PBS. The cells were collected by centrifugation, and 500 μL of protein lysate supplemented with a protease inhibitor cocktail (1:100) was added. The lysed cells were placed in 1.5‐mL tubes and fully lysed on ice for 30 minutes. The pellet was discarded by centrifugation, and the supernatant of the Pierce™ BCA Protein Assay Kit was quantified. Total protein (40 μg) was electrophoresed on a 15% SDS‐PAGE gel, electrotransferred onto a nitrocellulose membrane and identified by Western blot. The membranes were probed with rabbit anti‐MAL polyclonal antibody followed by secondary polyclonal HRP‐conjugated goat anti‐rabbit antibody. The blots were imaged using an AE‐1000 cool CCD image analyser.

### 2.14 Binding of mScarlet‐ETX and MAL to HEL cells or human erythrocytes

HEL cells were cultured, and erythrocytes were prepared as described above. The cells were incubated with rabbit anti‐MAL polyclonal antibody overnight at 4°C, then washed three times with 0.01 mol/L PBS, and incubated with FITC‐conjugated goat anti‐rabbit IgG（H + L）and mScarlet‐ETX (0.2 μmol/L) for 1 hours at room temperature. The cells were washed five times with 0.01 mol/L PBS and incubated with 4ʹ,6‐diamidino‐2‐phenylindole (DAPI). Finally, the stained cells were dropped onto a glass slide to mount, and the slides were imaged using a confocal microscope.

### 2.15 Propidium iodide (PI) staining

Cells were harvested and incubated with 0.2 μmol/L ETX at 37°C for 1 hours. The cells were washed once with PBS and resuspended in 200 μL of annexin V binding buffer containing 4 μL of 0.5 mg/mL PI. The stained cells were washed and resuspended with PBS and placed onto a glass slide for confocal microscopy.

### 2.16 Heptameric oligomerization

HEL cells (3 × 10^5^ cells/mL) were seeded in 6‐well plates and incubated at 37°C for 24 hours, washed with PBS and then incubated with 1 mL of His‐ETX proteins (0.2 μmol/L) for 60 min. The cells were washed 3 times with PBS, lysed by adding 500 μL of lysis buffer (50 mmol/L Tris‐HCl containing 1% Triton‐100 and 1% SDS) to each well and boiled at 100°C for 10 minutes. Total protein lysate was electrophoretically separated on a 10% SDS‐PAGE gel and transferred to a nitrocellulose membrane. The membrane was blocked with 5% bovine serum albumin for 1 hour at room temperature and then washed 3 times with 0.01 M PBS containing 0.05% Tween‐20 (PBST). The membrane was incubated with anti‐His monoclonal antibody overnight at 4°C, washed and incubated with HRP‐conjugated goat antimouse IgG antibody for 1 hour at room temperature. The membrane was washed five times with PBST and then subjected to exposure detection using an AE‐1000 cool CCD image analyser.

### 2.17 GST pull‐down

4 × 10^8^ cells were incubated with ETX (0.2 μmol/L) for 1 hour at room temperature and incubated with 1% Triton‐100 and protease inhibitor (1:100) in PBS buffer for 30 minutes at 4°C. The pellet was collected by centrifugation at 1200 *g* for 10 minutes, and lysed cells were added by lysis of 20 mL of buffer (50 mmol/L Tris‐HCl containing 1% Triton‐100). The GST or GST‐ETX suspensions were passed through a GSTrap HP purification column which was then washed with wash buffer A (1.8 mmol/L Na_2_HPO_4_.12H_2_O, 10 mmol/L NaH_2_PO_4_.2H_2_O, 140 mmol/L NaCl, 2.7 mmol/L KCl, 4 mmol/L DTT 1 L, pH 7.3). The cells membrane protein suspensions were flowed over the column and then eluted with solution B (50 mmol/L Tris‐HCl, 10 mmol/L Reduced glutathione, 4 mmol/L DTT, 1 L, pH 8.0). The eluted proteins were boiled for 10 minutes after adding 1 × protein loading buffer (125.0 mmol/L Tris‐Cl, 173.4 mmol/L SDS, 373.1 μmol/L Bromophenol Blue, 2.5 mL glycerine, 250 μL 2‐ME, 10 mL), electrophoresed on 12% SDS‐PAGE gels and transferred to nitrocellulose membranes. After blocking with 5% bovine serum albumin for 1 hour, the membranes were washed three times with 0.01 mol/L PBS containing 0.05% Tween‐20 (PBST) and incubated with anti‐GST monoclonal antibody or anti‐MAL polyclonal antibody overnight at 37°C, washed and incubated with HRP‐conjugated goat anti‐Rabbit IgG antibody for 1 hour at room temperature. The membrane was washed five times with PBST and then subjected to exposure detection using an imager.

### 2.18 Statistical analysis

Data analysis and statistics data are presented as mean ± SD. Each experiment was repeated at least three times. When multiple comparisons were made between groups, significant differences were calculated by one‐way analysis of variance (ANOVA) followed by Bonferroni test. A *P*‐value < 0.05 was considered to be a statistically significant.

## 3 RESULTS

### 3.1 ETX induces human erythrocyte shrinkage, swelling and lysis

**Figure 1:**
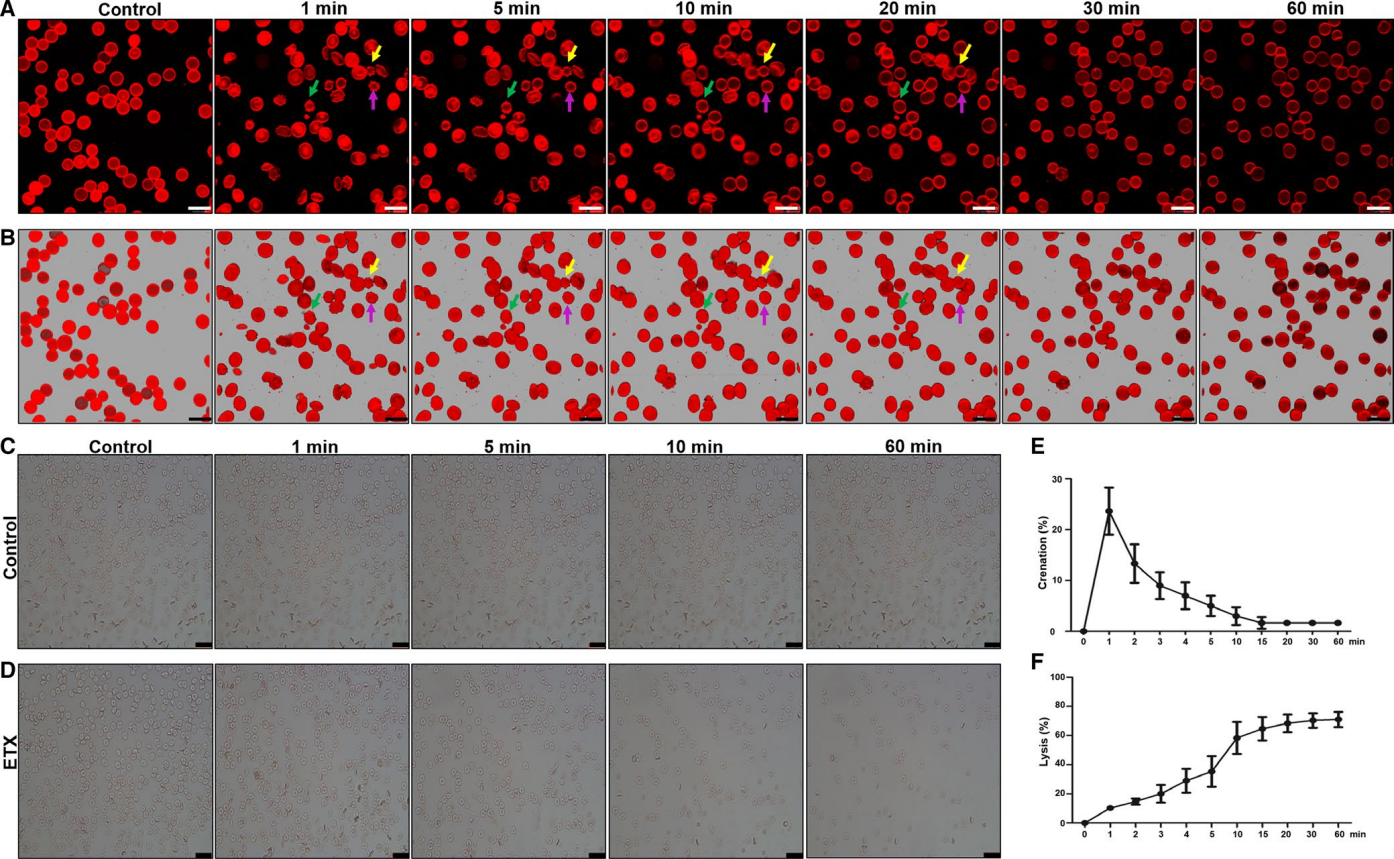
ETX induces human erythrocytes shrinkage, swelling and lysis. The cell membranes of human erythrocytes were stained with PKH26; then, the cells were incubated ETX and continuously imaged using confocal microscopy at 0, 1, 5, 10, 20 and 30 min. The arrows in the 2D (A) and 3D images (B) indicate that ETX causes contraction and swelling of human erythrocytes. The human erythrocytes were imaged after incubation with PBS (C) and ETX (0.2 μmol/L) (D) for 0, 1, 5, 10 and 60 minutes by light microscopy, respectively. E, F, Mean values displaying crenation and lysis over time in per cent cells. Values are mean ± SD, n = 3. A–B scale bar: 10 μm. C–D scale bar: 25 μm

Since ETX and *E coli* HlyA belong to β‐pore‐forming toxin family,^4^, ^19^ we first assessed whether ETX induced erythrocyte shrinkage, swelling and lysis in a manner similar to HlyA.^13^, ^20^ Here, we stained the cell membranes of human erythrocytes with PKH26, incubated the cells with ETX and imaged the cells using confocal microscopy. Within the first 1 minute, ETX induced the formation of spinous cells in erythrocytes, which may reflect shrinkage of the cells (Figure 1A,B). Similar results were obtained by light microscopy (Figure 1C), as compared to the negative control (Figure 1D). The cells swell after contraction, their morphology changes from spinous cells to spherocytes, and eventually rupture (Figure 1 A,B,D). Figure 1D shows images of human erythrocytes at 0, 1, 5, 10 and 60 minutes after ETX stimulation (0.2 μmol/L), with a gradual decrease in the number of red blood cells after 1 minute, confirming that ETX can cause erythrocyte lysis. Figure 1E,F shows the average data of contracted and lysed erythrocytes over time from three experiments. After incubation with ETX for 1 minute, the percentage of shrinking erythrocyte to total cells peaked. Over time, ETX causes a gradual increase in human erythrocyte lysis. It can be concluded that these changes in cellular appearance were a consequence of changes in cell volume.

### 3.2 ETX increases [Ca^2+^]_i_ in human erythrocytes

**Figure 2:**
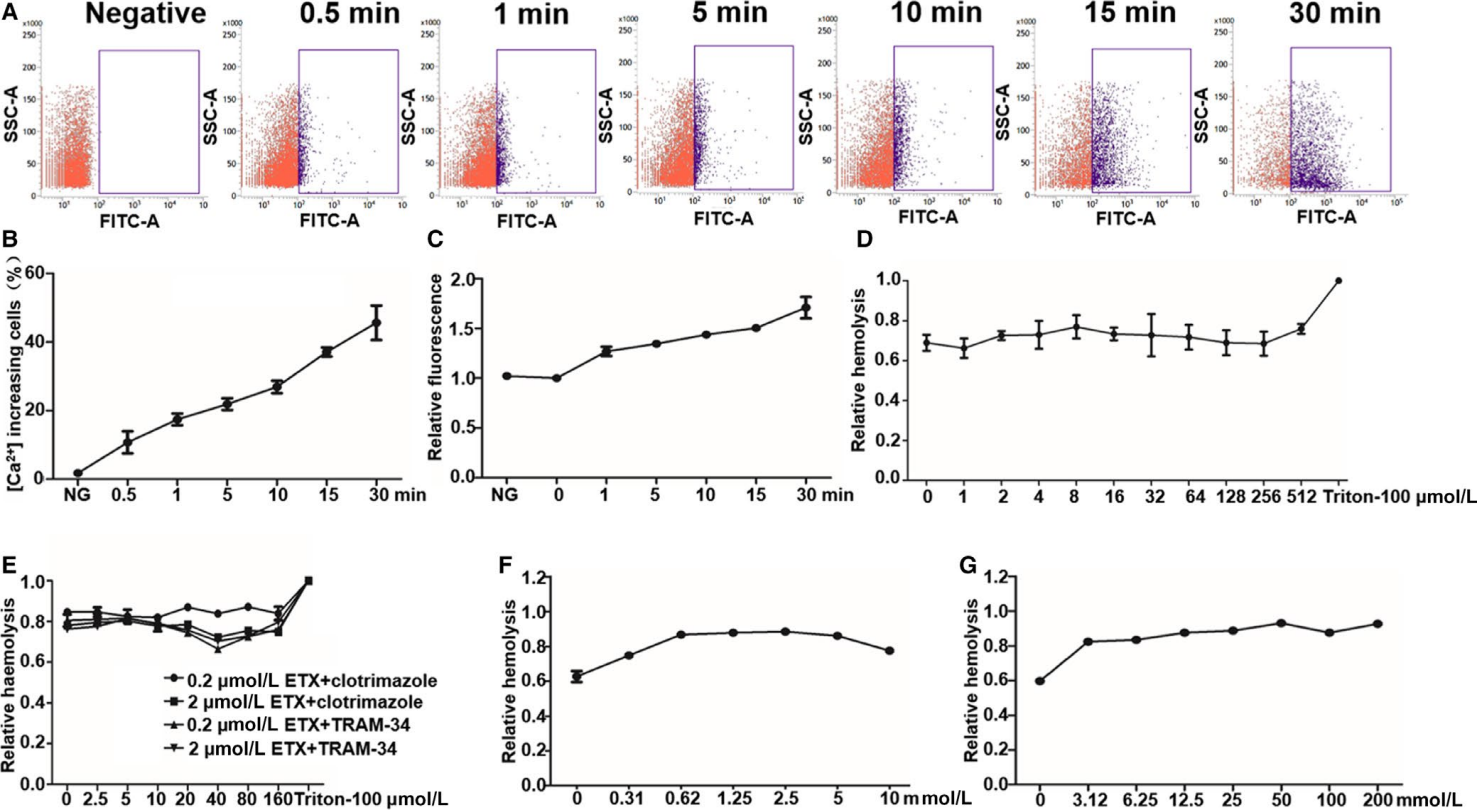
ETX increases [Ca^2+^]_i_ in human erythrocytes. A, Flow cytometry of erythrocytes loaded with the Ca^2+^‐sensitive dye fluo‐4 AM at 0, 1, 5, 10, 15 and 30 min after addition of ETX (0.2 μmol/L). B, Percentage of cells responding to ETX with an increase in [Ca^2+^]_i_ ([Ca^2+^]_i_‐increasing cells) at the indicated time of incubation. C, Change in fluo‐4 fluorescence from 100 cells 0‐30 min after ETX (0.2 μmol/L) was added. D, Different concentrations (1, 2, 4, 8, 16, 32, 64, 128, 256 and 512 μmol/L) of BAPTA‐AM did not affect ETX‐induced haemolysis. E, The effect of ETX on the KCa3.1 channel. Cells were incubated with ETX (0.2 and 2 μmol/L) for 60 min in the presence or absence of increasing concentrations of clotrimazole and TRAM‐34, two KCa3.1 channel antagonist. F, EGTA‐buffered Ca^2+^ free saline potentiates ETX‐induced haemolysis. Erythrocytes were incubated with 0.2 μmol/L ETX for 60 min at 37°C. G, Increasing the extracellular concentration of K^+^ potentiates ETX‐induced haemolysis, erythrocytes were incubated with 0.2 μmol/L ETX for 60 min at 37°C

ETX forming a heptameric toxin pore is a β‐barrel pore characterized by an arrangement of 14 amphoteric β‐strands.^4^ The significant initial ETX‐induced shrinkage of erythrocytes implied that ion efflux early during this process exceeded ion influx. To test whether the contraction caused by ETX has a similar mechanism, we verified if ETX triggered a change in [Ca^2+^]_i_. Figure 2A displays representative images of erythrocytes incorporating fluo‐4 AM (Ca^2+^‐sensitive dye) at 0.5, 1, 5, 10, 15 and 30 minutes after addition of ETX (0.2 μmol/L). Within 0.5 minutes, we observed a significant increase in the number of erythrocytes attached to fluo‐4 indicating an increased [Ca^2+^]_i_, which occurred before cell shrinkage. The percentage of ETX‐induced [Ca^2+^]_i_ cells increased with longer incubation times (Figure 2B). Similarly, Figure 2C displays the mean change in fluo‐4 fluorescence from 100 cells for 0‐30 minutes of incubation with ETX (0.2 μmol/L). However, addition of a calcium ion‐binding chelator (BAPTA‐AM) did not inhibit ETX‐induced haemolysis (Figure 2D). Subsequently, we added calcium ion‐binding chelating agent (BAPTA‐AM) to the calcium‐free buffer without inhibiting ETX‐induced haemolysis similar to Figure 2D (data not shown). These data indicate that ETX causes influx of calcium ions, but the influx of calcium ions is not important for ETX‐induced haemolysis.

The volume reduction caused by ETX showed that the formation of pores induced the flow out of net ions rather than flow in. As the concentration of K^+^ was the most important intracellular cation, there may be an outflow of K^+^ during the shrinkage.^21^ We first tested that if the shrinkage was caused by Ca^2+^‐activated K^+^ efflux, but clotrimazole and TRAM‐34 (Ca^2+^‐activated K^+^ channel (K_Ca_3.1 channel) blocker) at concentrations of 0‐160 mmol/L did not affect ETX‐induced haemolysis (Figure 2E), which is different from other pore formers. However, a significant increase in the concentration of [Ca^2+^]_i_ with ETX treatment was supposed to activated K_Ca_3.1 channel, and reduction of Ca^2+^ concentration by Ca^2+^‐free EGTA‐containing saline significantly enhanced ETX‐induced haemolysis (Figure 2F), which suggested that the initial contraction of the cells is at least partially caused by Ca^2+^‐activated K^+^ efflux. Furthermore, the increase of concentration of extracellular K^+^ could gradually enhanced ETX‐induced haemolysis (Figure 2G), indicating that ETX caused haemolysis of erythrocytes through the imbalance of K^+^ efflux.

### 3.3 ETX triggers PS exposure in the outer leaflet of the erythrocyte membrane

**Figure 3:**
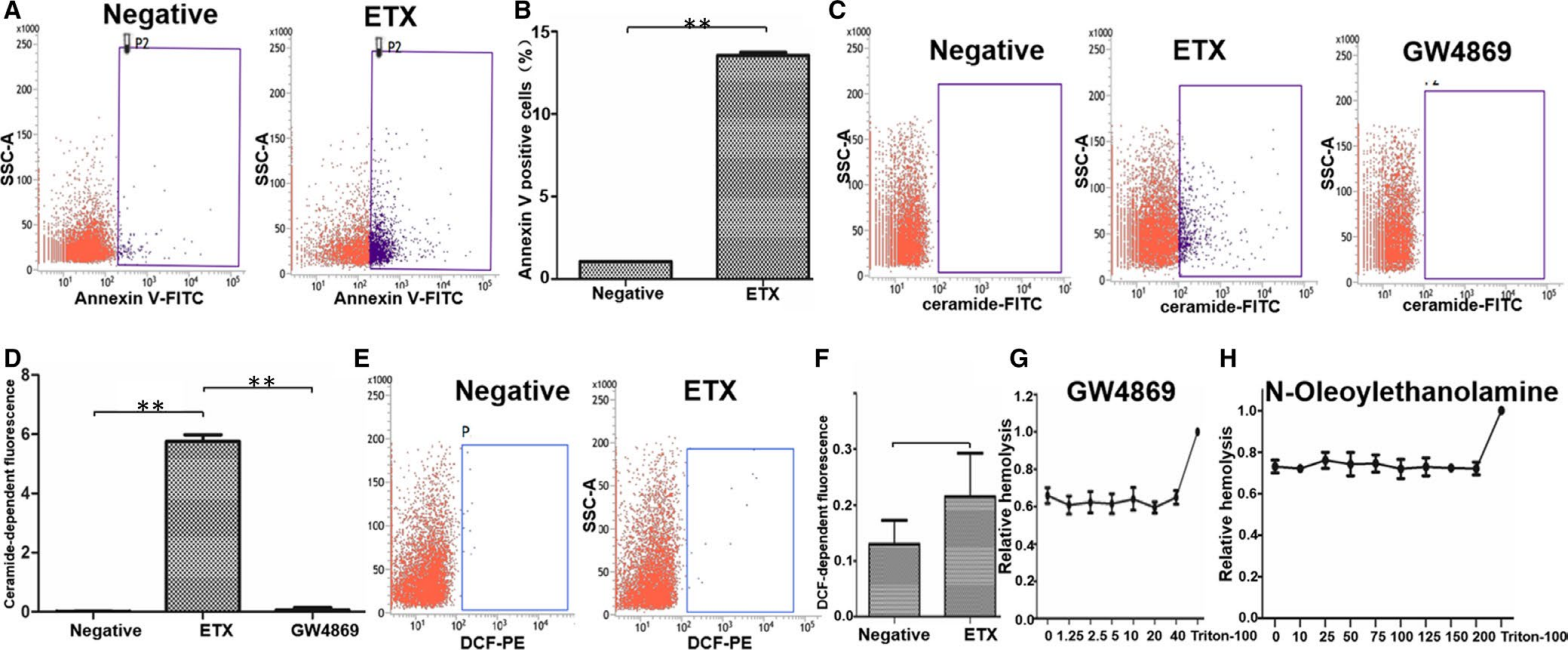
ETX triggers PS exposure in the outer leaflet of the erythrocyte membrane. A, Flow cytometry showed that ~14% of human erythrocytes had significant annexin V staining after ETX (0.2 μmol/L) incubation. B, Proportion of human erythrocytes stained with annexin V after ETX (0.2 μmol/L) incubation. Values represent means ± SD (n = 3). ETX enhanced erythrocyte ceramide abundance, but did not trigger production of reactive oxygen species. C, Exposure to ETX (0.2 μmol/L) increased ceramide formation in erythrocytes, and GW4869 inhibited ETX‐induced increases in ceramide abundance. D, ETX induced a 5.8% increase in ceramide concentration compared to the negative control group. E, F, Flow cytometry analysis of DCF‐dependent fluorescence showing that ETX treatment does not stimulate ROS production in human erythrocytes. (g) Different concentrations (0‐40 μmol/L) of GW4869 did not inhibit ETX‐induced haemolysis of human erythrocytes. (h) Different concentrations (0‐200 μmol/L) of N‐oleoylethanolamine did not inhibit ETX‐induced haemolysis of human erythrocytes

Increased [Ca^2+^]_i_ levels trigger an early senescent response in erythrocytes, including exposure of PS.^22^ Therefore, we explored whether ETX could cause PS exposure and FITC‐conjugated annexin V was used for this assay, which has high affinity for PS. We incubated red blood cells with 0.2 μM ETX and observed ~14% of cells with significant annexin V staining (Figure 3A,B). These data demonstrated that ETX triggered PS exposure in the outer leaflet of the erythrocyte membrane.

### 3.4 ETX enhanced erythrocyte ceramide abundance, but did not trigger production of reactive oxygen species

We further explored other mechanisms involved in ETX‐induced erythrocyte death. A previous study showed that PS exposure was related to ceramide accumulation.^12^, ^23^ Additionally, ETX oligomer formation is induced by activation of neutral sphingomyelinase and production of ceramide.^24^ Thus, we speculated that the toxic effects of ETX might be related to sphingomyelinase activity. We therefore examined whether ETX treatment influenced sphingomyelinase activation, which mediates ceramide formation in erythrocytes. Exposure to 0.2 μM ETX increased ceramide formation in erythrocytes (Figure 3C and D). However, inhibitors of neutral sphingomyelinase (GW4869) and the neuraminidase inhibitor (N‐oleoylethanolamine) did not inhibit ETX‐induced haemolysis (Figure 3G and H). We further investigated the effect of GW4869 on ETX‐induced ceramide production. Figure 3C showed GW4869 inhibited ETX‐induced ceramide formation. These data indicate that ETX‐induced haemolysis enhanced ceramide abundance, but ceramide formation is not important for ETX‐induced haemolysis. Next, we measured dihydrodichlorofluorescein (DCF)‐dependent fluorescence by flow cytometry to assess if ETX induced ROS production in human erythrocytes. ETX treatment did not stimulate ROS generation in erythrocytes (Figure 3E and F), suggesting that ETX‐induced haemolysis was not paralleled by redox imbalances.

### 3.5 ETX cytotoxicity in HEL cells requires MAL receptors

**Figure 4:**
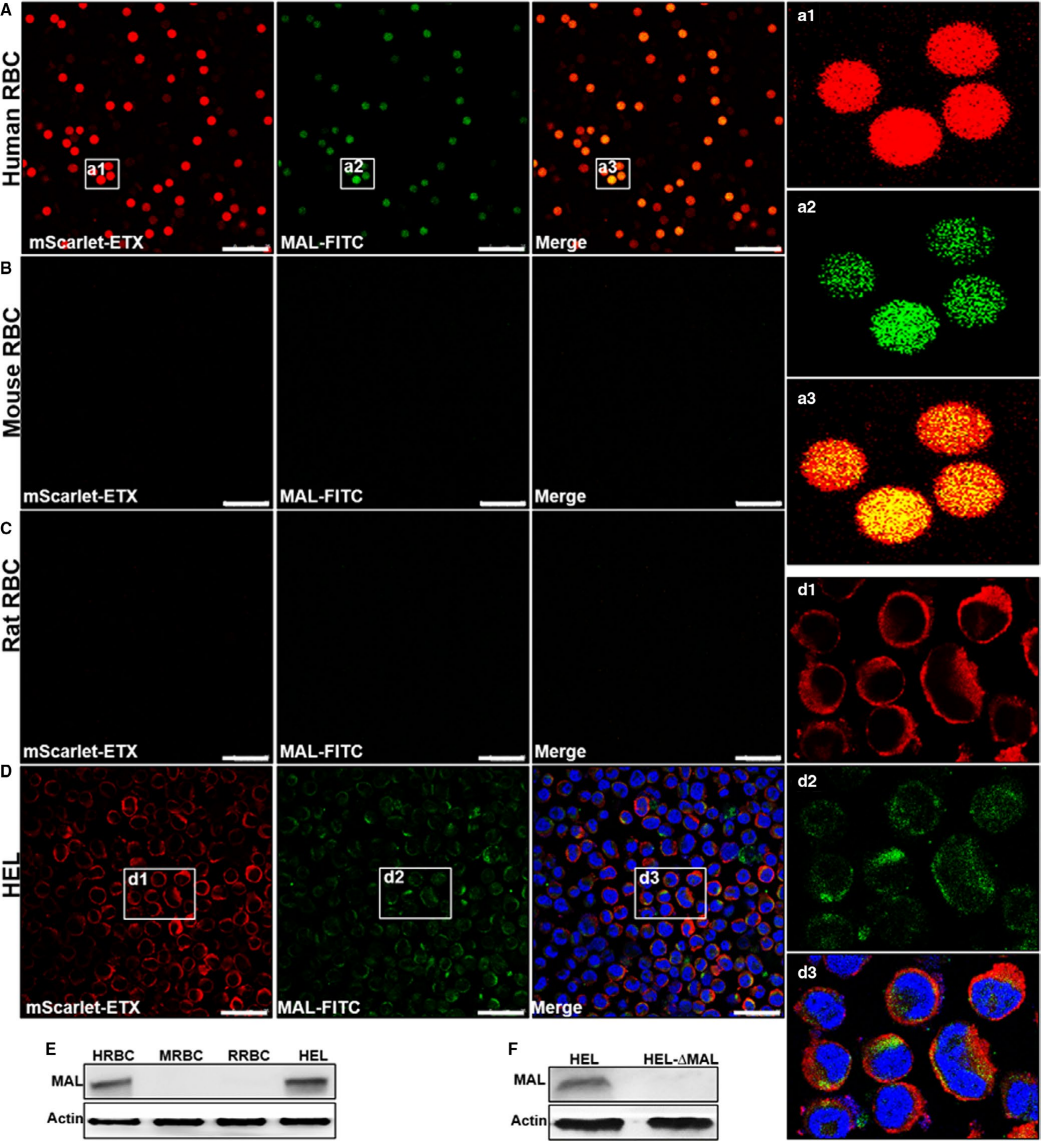
Expression of MAL protein on the cell membranes of human erythrocytes and HEL cells. MAL co‐localized with ETX‐mScarlet on the cell membrane, but neither expression of MAL protein nor binding of ETX‐mScarlet was observed for mouse and rat erythrocytes. A–D, Confocal imaging revealed that MAL protein was expressed on the cell membranes of human but not mouse or rat erythrocytes as well as on HEL cells. E, Expression of MAL protein on human erythrocytes and HEL cells was confirmed by Western blotting. F, MAL protein expression was silenced via lentivirus‐mediated RNA interference in HEL cells, and MAL protein expression in the resulting HEL‐ΔMAL clones was assessed by Western blotting. Regions (a1‐a3 and d1‐d3) framed in a and d are shown at a higher magnification to illustrate co‐staining details. Images in a1, a2, d1, d2 are shown in separate fluorescence channels and as an overlay in a3 and d3. Noticeably, a1‐a3 and d1‐d3 display that the expression of MAL is co‐localized with the binding of ETX‐mScarlet. A–D scale bar: 25 μm

We have previously found that ETX is highly specific for human erythrocytes, but it has no toxic effects on erythrocytes from other species.^9^ We incubated mouse, rat and human erythrocytes with mScarlet‐ETX and found that mScarlet‐ETX bound to human erythrocytes (Figure 4A) but not to mouse (Figure 4B) and rat erythrocytes (Figure 4C). These findings suggest that ETX‐binding receptors are present on human erythrocytes but absent in mouse and rat erythrocytes.

MAL has recently been found to be involved in the cytotoxicity of ETX.^25^ We stained human, mouse and rat erythrocytes and found that MAL was expressed in human erythrocytes (Figure 4A), but not in mouse (Figure 4B) and rat erythrocytes (Figure 4C). We also confirmed the expression of MAL receptors only in human red blood cells by Western blotting (Figure 4E). Confocal microscopy revealed that expression of MAL proteins was mostly localized to the plasma membrane. Most mScarlet‐ETX binds around the cell membrane (Figure 4A and D).

**Figure 5:**
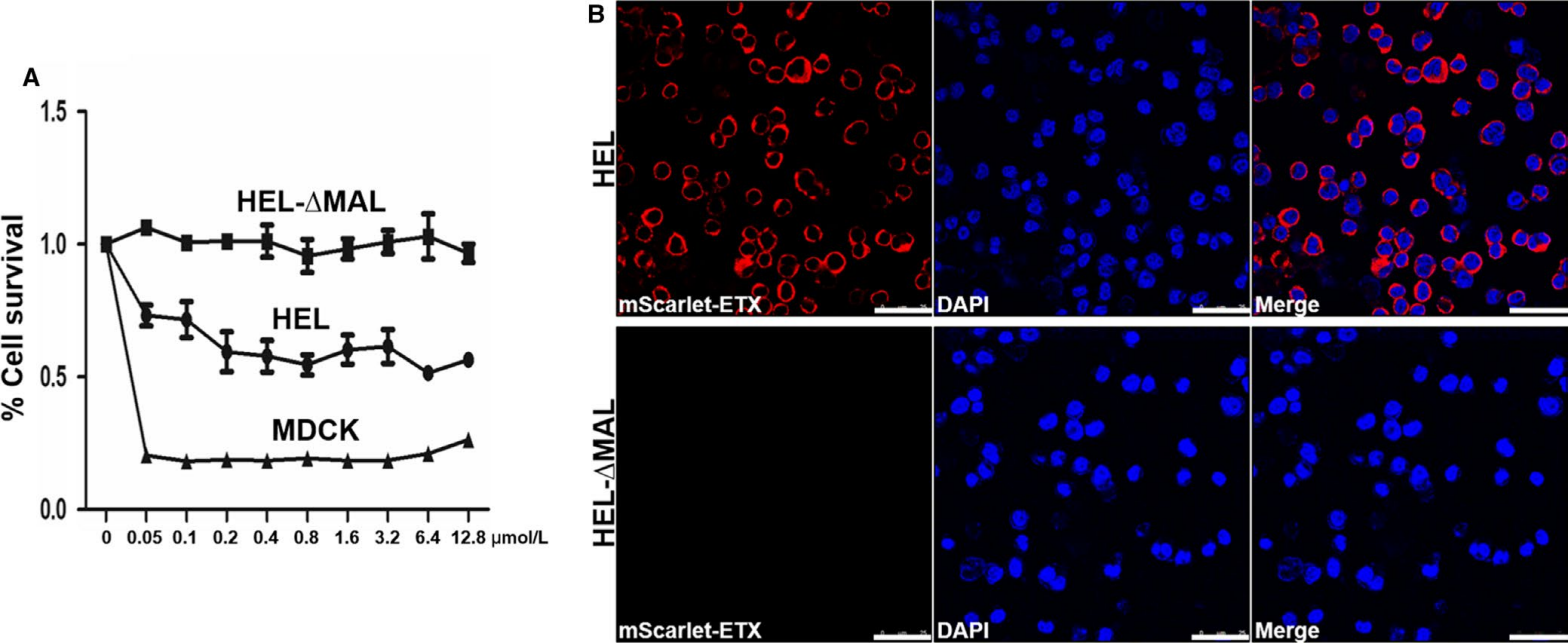
ETX binding and cytotoxicity depend on MAL expression. A, A HEL‐ΔMAL clone that did not express MAL protein showed no ETX cytotoxicity by MTS colorimetric assay. B, Confocal images of fixed HEL and HEL‐∆MAL cells treated with 2 μmol/L mScarlet‐ETX for 60 min. Cells were washed and counter‐stained with DAPI (blue) to stain nuclei. The presence of mScarlet‐ETX around the plasma membrane of HEL cells was evident. In contrast, there was no detectable mScarlet‐ETX signal in the HEL‐∆MAL cells. B scale bar: 25 μm

Since inhibitors of MAL receptors have not been identified and it is not possible to knock out the *mal* gene in human erythrocytes, HEL cells were used as surrogates of human erythrocytes. HEL cells represent the erythroblastic stage of differentiation of hematopoietic cells and can differentiate into megakaryocytes, macrophages or erythrocytes. We confirmed that ETX caused HEL cell death and that these cells expressed MAL protein (Figure 4E). Most of the mScarlet‐ETX was located around HEL cells and co‐localized with MAL proteins (Figure 4D). To demonstrate that MAL expression is involved in the cytotoxic effects of ETX, MAL was depleted in HEL cells via lentivirus‐mediated RNA interference. Several MAL‐deleted clones (HEL‐∆MAL) were obtained, and the toxic effects of ETX on HEL‐∆MAL were analysed. Silencing of MAL expression in HEL‐∆MAL clones was confirmed by Western blotting (Figure 4F). Cytotoxicity assays (MTS colorimetric assays) of HEL‐∆MAL clones revealed ETX did not have a cytotoxic effect when MAL protein was not expressed (Figure 5A). Furthermore, absence of ETX binding to HEL‐∆MAL clones was demonstrated by confocal microscopy (Figure 5B). These results clearly indicate that the absence of expression of MAL protein directly impairs the binding of ETX to cells and the cytotoxic effects of ETX.

### 3.6 MAL protein is required for ETX‐induced oligomeric complex and pore formation

**Figure 6:**
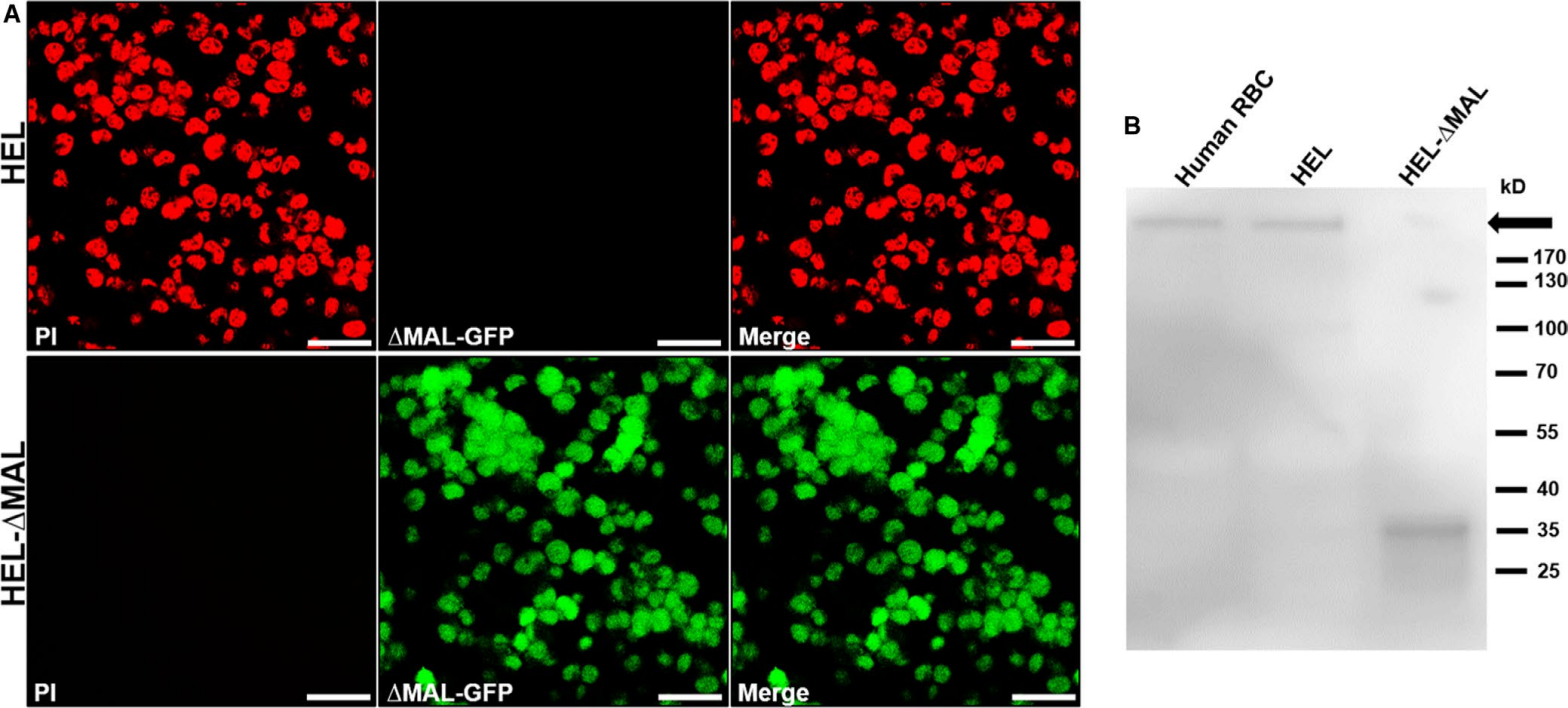
No ETX‐forming oligomeric complex and pores were detected in HEL‐ΔMAL cells. A, HEL and HEL‐∆MAL cells were treated with 0.2 μmol/L ETX at 37°C for 1 hours. The cells were washed once with PBS, and 0.5 mg/mL PI was added for 15 min. ETX caused PI to stain HEL cell nuclei but not those of HEL‐∆MAL clones, indicating that MAL plays a key role in the pore‐forming effect of ETX in HEL cells. B, Human erythrocytes, HEL and HEL‐∆MAL cells were treated with 0.2 μM ETX for 60 min. Western blotting of cell lysates using anti‐ETX antibody revealed oligomeric complexes (>170 kDa, black arrowhead) in human erythrocytes and HEL cells. No oligomeric complexes were observed in HEL‐∆MAL cells. A scale bar: 25 μm

The cytotoxic effect of ETX occurs through binding to specific receptors on target cells, followed by oligomerization and pore formation, permeabilizing cell membranes and allowing the diffusion of ions and other molecules up to 2.3 kDa in size.^8^, ^26^ PI staining was used to assess whether ETX could induce pore formation in HEL and HEL‐ΔMAL cells. The results confirmed that ETX induced pore formation in HEL cells but not in HEL‐ΔMAL cells (Figure 6A). Western blot analysis showed that there was no membrane complex formation after incubation of HEL‐∆MAL cells with ETX (Figure 6B). These results suggested that MAL protein is involved in ETX‐induced pore and oligomeric complex formation.

### 3.7 ETX directly interacts with MAL receptors in the cell membranes of human erythrocytes

**Figure 7:**
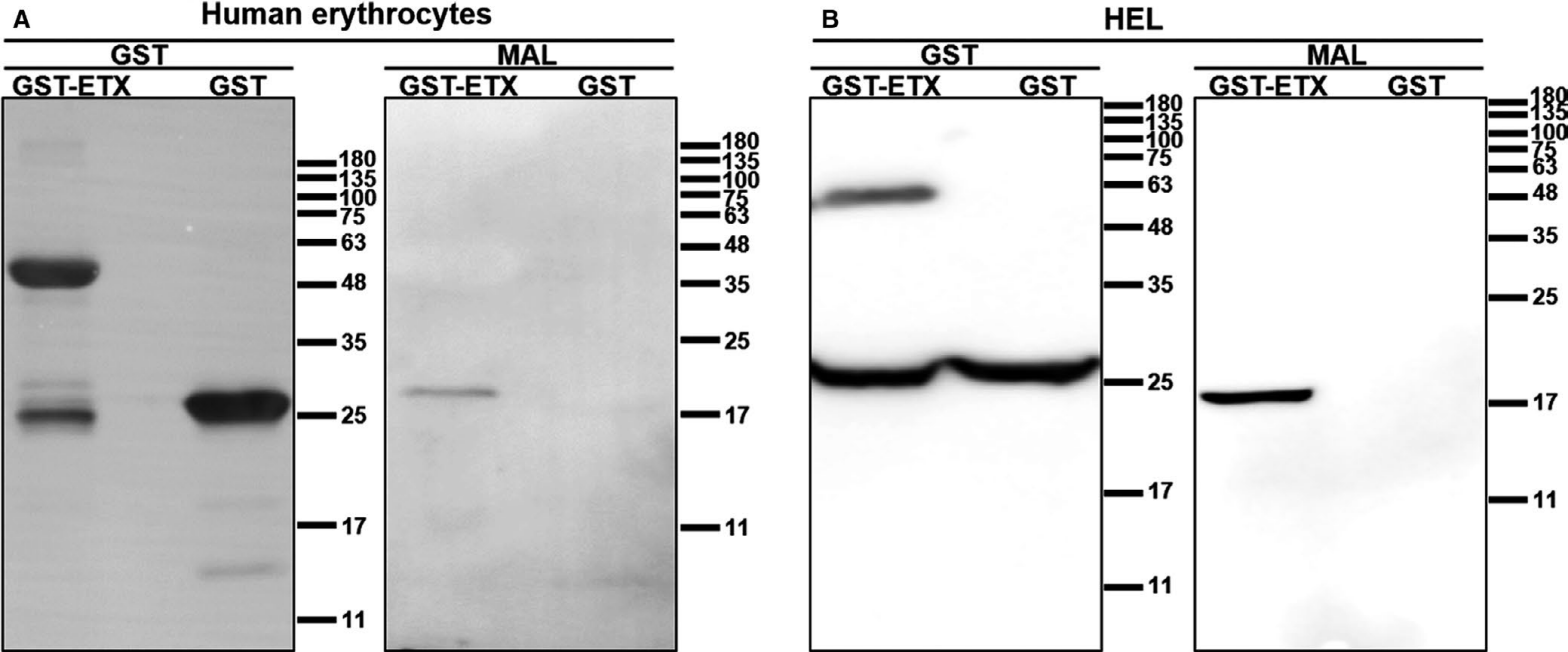
ETX interacts directly with MAL receptors in the cell membranes of human erythrocytes. We extracted the membrane proteins of human erythrocytes and passed them through GST and GST‐ETX purification columns. We then analysed the presence of GST and MAL in the purified proteins of human erythrocytes (A) and HEL cells (B) by Western blotting

The above results confirmed that MAL receptors play an important role in ETX‐induced cytotoxicity of HEL cells and that MAL protein expression was required in human erythrocytes and HEL cells for ETX binding. In order to directly observe whether ETX can bind to MAL receptors, we passed lysates of human erythrocyte and HEL cell membranes through a GST affinity column. Western blotting of the purified protein revealed that ETX can directly bind MAL receptors in vitro (Figure 7). These experiments clearly demonstrated that ETX can interact with MAL receptors on human erythrocytes.

## 4 DISCUSSION

ETX has been found to form toxin pores on target cell membranes since 2001.^8^ Clinically, it has been reported that in patients infected with *C perfringens*, large‐scale intravascular haemolysis occurs, sometimes with severe anaemia.^27^, ^28^ The mechanism of causing severe anaemia during *C perfringens* infection has not been elucidated, but it is considered that erythrocyte haemolysis caused by ETX may be necessary for the treatment of *C perfringens* infection. Recently, we found that ETX is highly specific for human erythrocytes but not sensitive to erythrocytes of other species (such as sheep and goats).^11^ However, the mechanism of haemolysis caused by ETX remains unclear. In this study, we further studied and defined the mechanism by which ETX causes erythrocyte lysis.

In the present study, it was found that ETX caused a significant decrease in human erythrocyte volume, and then swelled and lysis. The initial volume reduction is caused by the inflow of Ca^2+^ and the outflow of K^+^. In addition, ETX also triggers PS exposure on the cell membrane and increases the abundance of ceramide. Mechanistically, intracellular Ca^2+^ activity is a crucial participant in eryptosis signalling.^29^ Our results indicate that the first event after adding ETX is an increase in [Ca^2+^]_i_ (Figure 2). BAPTA‐AM, an intracellular calcium chelator, did not inhibit haemolysis caused by ETX. We hypothesize that ETX causes erythrocyte Ca^2+^ influx, but that changes in Ca^2+^ concentration is not necessary for haemolysis. Thus, a plausible explanation for lack of an effect of [Ca^2+^]_i_ on haemolysis could be that ETX triggered very slow changes in [Ca^2+^]_i_. Activation of calcium ion influx pathways may not potentiate haemolysis and thus may not be an absolute requirement for lysis to occur. Ca^2+^ further activates the K_Ca_3.1 channel, resulting in loss of KCl and cell shrinkage. Eryptosis signalling involves activation of the cation channel and entry of Ca^2+^, as well as subsequent activation of sphingomyelinase and production of ceramide.^12^ We found that ETX could trigger an increase in ceramide abundance in human erythrocytes using flow cytometry (Figure 3C). Ceramide enhances the Ca^2+^ sensitivity of cell membranes, which is similar to increasing cytosolic Ca^2+^ activity and increases PS exposure.^30^ Studies have confirmed that GW4869 as well as silencing of nSMase with siRNAs inhibited ETX‐induced cytotoxicity in MDCK and ACHN cells.^24^ However, our data showed that ETX can enhance erythrocyte ceramide abundance and that the neutral sphingomyelinase inhibitor (GW4869) could inhibit ceramide formation induced by ETX. However, neither GW4869 nor N‐oleoylethanolamine inhibited ETX‐induced haemolysis. Taken together, the data suggested that ETX did activate nSMase, but that nSMase activation played a negligible role in haemolysis caused by ETX. We show that ETX increases PS exposure in erythrocytes during haemolysis. This early response to cell contraction and PS exposure is similar to the initial phase of nucleated cell apoptosis. Cell shrinkage can identify and remove these cells prior to lysis, free of immediate lysis. In this case, macrophages can specifically detect PS‐exposed cells to eliminate circulating senescent erythrocytes.^31^, ^32^ Therefore, cell shrinkage and PS exposure on the cell membrane induced by ETX may be essential for the identification and clearance of damaged erythrocytes from the blood circulation, thereby avoiding intravascular haemolysis during fatal enterotoxaemia caused by bacteria producing ETX.^33^

Using mScarlet‐ETX fluorescent staining, we confirmed our previous finding that ETX is highly specific for human erythrocytes.^11^ Taken together, our results indicate that the ETX receptor is only present on the surface of human erythrocytes. MAL has recently been associated with ETX‐induced cell death in oligodendrocytes,^15^ human T lymphocytes^16^ and polarized epithelial cells.^25^ We confirmed the high expression of MAL receptor in human erythrocytes by co‐staining with anti‐CD235a antibody (Figure Fig S1) and showed that the MAL protein was expressed only in the membrane of human erythrocytes by immunofluorescence and Western blot. mScarlet‐ETX bound to human but not to mouse or rat erythrocytes, and co‐localized with MAL on human erythrocyte cell membranes. Our previous data also indicated that ETX only caused human erythrocytes haemolysis, but had no effect on mouse and rat erythrocytes. Based on the association between haemolysis and MAL expression, we conclude that MAL receptors play an important role in erythrocyte haemolysis caused by ETX. We further investigated the effects of ETX in MAL‐deficient HEL cells and found that HEL cells also expressed MAL receptors, which co‐localized with mScarlet‐ETX on the cell membrane, and that ETX had a cytotoxic effect on HEL cells but not on MAL‐deficient HEL cells. In addition, ETX did not bind MAL‐deficient HEL cells and could not form pores or oligomeric complexes on the membranes of these cells. Together, these dates support the hypothesis that MAL is the cellular receptor for ETX. We further confirmed a direct interaction between ETX and MAL receptors on human erythrocytes and HEL cells in vitro using a pull‐down assay (Figure 7). As far as we know, this is the first time showing a direct bind of ETX to MAL proteins in vitro. All of these observations support the notion that the MAL protein is the likely receptor for ETX.

In conclusion, our results underscore that ETX can only induce haemolysis of human erythrocytes, but it cannot induce haemolysis of erythrocytes in animals. Thus, ETX is potentially toxic to humans and may cause disease in humans to be more severe than in animals. ETX causes haemolysis is a process of volume change in human erythrocytes with an increase in PS exposure. This mechanism may be critical for the clearance of ETX‐inserted erythrocytes in blood circulation after *C perfringens* infection. In addition, given that MAL receptors play an important role in ETX binding, ETX‐induced cytotoxicity, polymer formation and pore formation. Together with our data confirming an in vitro interaction between ETX and MAL, we conclude that MAL is likely the receptor for ETX, this protein represents a potential target for treatment of ETX‐induced diseases.

## CONFLICT OF INTEREST

No potential conflict of interest was reported by the author.

## AUTHOR CONTRIBUTIONS

ZG and WX were responsible for the design and primary technical process, conducted the experiments, collected and analysed data, and wrote the manuscript. JH and LK helped perform the main experiments. SG and YY participated in the flow cytometry. YL and JW participated in cell experiments and post‐examination. WX and JW collectively oversaw the collection of data and data interpretation and revised the manuscript.

## Supporting information


**Fig S1**

## Data Availability

The data used to support the findings of this study are included in the article.
