# Supplementary figures and images for: Clostridium perfringens epsilon toxin binds to erythrocyte MAL receptors and triggers phosphatidylserine exposure
Source: J Cell Mol Med. 2020 May 28;24(13):7341–52. doi: 10.1111/jcmm.15315 (PMC7339222; doi:10.1111/jcmm.15315)

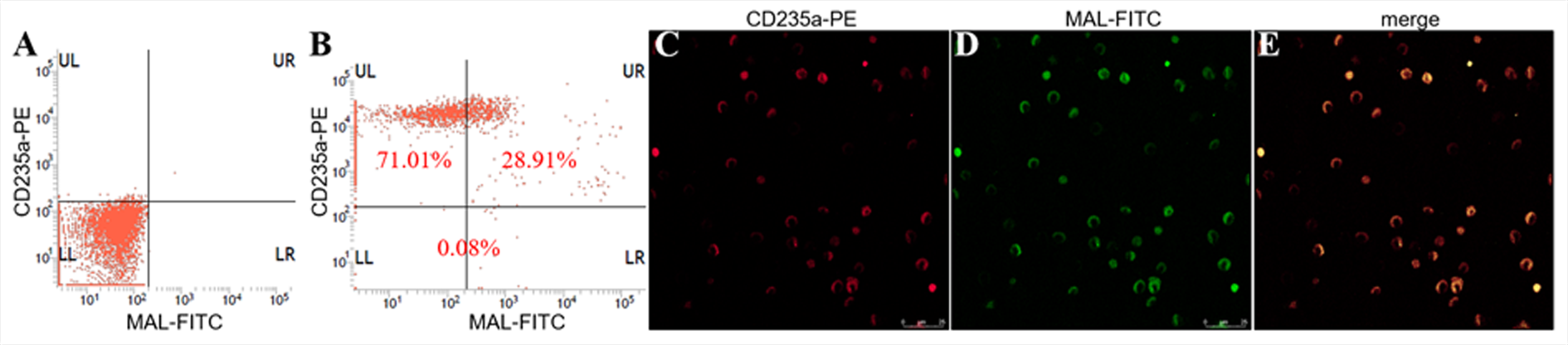

Supplement: Supplementary file 1 — Fig S1 [file JCMM-24-7341-s001.tif]
